# Case study of semaglutide and cardiovascular outcomes: An application of the C*ausal Roadmap* to a hybrid design for augmenting an RCT control arm with real-world data

**DOI:** 10.1017/cts.2023.656

**Published:** 2023-10-23

**Authors:** Lauren E. Dang, Edwin Fong, Jens Magelund Tarp, Kim Katrine Bjerring Clemmensen, Henrik Ravn, Kajsa Kvist, John B. Buse, Mark van der Laan, Maya Petersen

**Affiliations:** 1 Department of Biostatistics, University of California, Berkeley, CA, USA; 2 Novo Nordisk, Søborg, Denmark; 3 Division of Endocrinology, Department of Medicine, University of North Carolina, Chapel Hill, NC, USA

**Keywords:** Causal inference, real-world evidence, hybrid study designs, diabetes, cardiovascular outcomes

## Abstract

**Introduction:**

Increasing interest in real-world evidence has fueled the development of study designs incorporating real-world data (RWD). Using the *Causal Roadmap*, we specify three designs to evaluate the difference in risk of major adverse cardiovascular events (MACE) with oral semaglutide versus standard-of-care: (1) the actual sequence of non-inferiority and superiority randomized controlled trials (RCTs), (2) a single RCT, and (3) a hybrid randomized-external data study.

**Methods:**

The hybrid design considers integration of the PIONEER 6 RCT with RWD controls using the experiment-selector cross-validated targeted maximum likelihood estimator. We evaluate 95% confidence interval coverage, power, and average patient time during which participants would be precluded from receiving a glucagon-like peptide-1 receptor agonist (GLP1-RA) for each design using simulations. Finally, we estimate the effect of oral semaglutide on MACE for the hybrid PIONEER 6-RWD analysis.

**Results:**

In simulations, Designs 1 and 2 performed similarly. The tradeoff between decreased coverage and patient time without the possibility of a GLP1-RA for Designs 1 and 3 depended on the simulated bias. In real data analysis using Design 3, external controls were integrated in 84% of cross-validation folds, resulting in an estimated risk difference of –1.53%-points (95% CI –2.75%-points to –0.30%-points).

**Conclusions:**

The *Causal Roadmap* helps investigators to minimize potential bias in studies using RWD and to quantify tradeoffs between study designs. The simulation results help to interpret the level of evidence provided by the real data analysis in support of the superiority of oral semaglutide versus standard-of-care for cardiovascular risk reduction.

## Introduction

Regulatory agencies around the world have increasingly considered how studies that incorporate real-world data (RWD) might inform the regulatory approval process [1]. One particular class of studies considers integration of trial data with RWD or other external data sources [2]. For example, single-arm trials compare a treatment with an external control group. Hybrid studies randomize participants to active treatment or control and aim to augment one or both arms of the trial with external data. A third use of external data is to compare treatments evaluated in different trials, although the comparator arms in these trials may have evaluated the same drug [3].

These study designs require special considerations. First, each design relies on different causal identification assumptions – i.e., assumptions about the underlying trial and/or real-world processes that generate the data that will be used to estimate a treatment effect. For many designs, these assumptions are not testable, but the validity of inferences about treatment effects nonetheless relies on them. Second, while single-arm trials may be analyzed using traditional statistical estimators, special estimators have been developed for analyzing data from the second two types of external data designs [3,4]. If the same treatment (or control) is evaluated in the randomized and external data, bias due to violations of identification assumptions may be estimated to determine whether to include external data in the analysis [5]. Hybrid designs thus provide greater protection against biased conclusions than purely observational designs, but because bias is estimated, these designs cannot guarantee 95% confidence interval coverage for the true effect [5], as would be expected of a traditional randomized controlled trial (RCT).

The purpose of this paper is to demonstrate how to apply a *Causal Roadmap* [7–9], described in the companion paper [10], to the design and analysis of a hybrid randomized-RWD study using a case study of the effect of semaglutide on the risk of major adverse cardiovascular events (MACE). We discuss threats to causal inference when RWD is integrated with RCT data. We also provide a detailed demonstration of Step 7 of the *Causal Roadmap*, which involves comparing alternative study designs using simulations. Our discussion of this complex example aims to facilitate comprehension of the Roadmap steps described in the companion paper.

## Case Study

Semaglutide, a glucagon-like peptide-1 receptor agonist (GLP-1RA), was developed as an antihyperglycemic agent and has been shown to improve multiple health outcomes for patients with type 2 diabetes mellitus (T2DM). For example, in the SUSTAIN-6 trial, injectable semaglutide decreased rates of MACE (defined as death from cardiovascular causes or nonfatal stroke or myocardial infarction (MI)) compared to placebo in patients with high cardiovascular risk (hazard ratio (HR) 0.74, 95% confidence interval (CI) 0.58–0.95) [11]. Subsequently, the United States Food and Drug Administration (FDA) approved injectable semaglutide to reduce cardiovascular risk for adults with T2DM and cardiovascular disease.

Oral semaglutide was later developed and shown to decrease glycated hemoglobin (HbA1c) and body weight compared to placebo [12] and multiple medications [13–15]. To satisfy a pre-approval regulatory requirement for demonstrating cardiovascular safety, the PIONEER 6 RCT evaluated non-inferiority of oral semaglutide versus placebo for the primary outcome of MACE, with an estimated HR of 0.79 (95% CI, 0.57–1.11) [16]. To establish whether oral semaglutide is superior to placebo for the prevention of MACE, the larger SOUL trial began in 2019 [17].

A superiority RCT is a standard choice for evaluating the effect of interest, yet RCTs may also have downsides. For example, clinicians treating placebo-arm patients are directed not to prescribe medications of the same class as the treatment under investigation [17–19]. Yet in 2019, a joint statement by the American Diabetes Association and the European Association for the Study of Diabetes emphasized that “for patients with type 2 diabetes and established atherosclerotic [cardiovascular] disease … the level of evidence for MACE benefit is greatest for GLP-1 receptor agonists” [20]. Although none of the trial participants were taking a GLP-1RA at baseline [17], would it not be better for the participants in the placebo arm of SOUL if they were allowed to start a GLP-1RA? This question led us to ask whether a hybrid trial design incorporating RWD could decrease the amount of participant time during which commencement of a GLP1-RA is precluded. Yet this potential benefit must be weighed against the known risk that confidence interval coverage may fall below 95% (or type 1 error may increase) in a hybrid design, depending on the magnitude of bias introduced by the external data [21–24].

To evaluate these potential tradeoffs, we use the *Causal Roadmap* [7–10] to compare a traditional program of RCTs to a hybrid study integrating data from the PIONEER 6 non-inferiority trial with RWD controls through simulations that mimic these true experiments. We then present results of the real hybrid analysis of PIONEER 6 and RWD for the estimated difference in the risk of a combined outcome of first MI, stroke, or all-cause death with oral semaglutide versus standard-of-care (without a GLP1-RA).

## Materials and Methods

**Figure 1.**
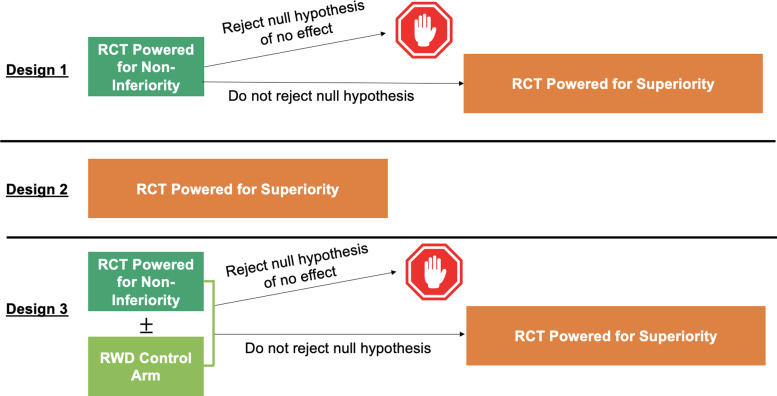
Diagram of study Designs 1–3. RCT=randomized controlled trial. RWD=real-world data.

Table 1 describes design and analysis plans for three potential study designs for evaluating this question, using the list of *Causal Roadmap* steps found in the companion article [10]. As depicted in Fig. 1, Design 1 is based on what truly occurred – a non-inferiority trial was run to demonstrate cardiovascular safety of oral semaglutide (PIONEER 6), after which, due to results that were promising but non-significant for superiority, a superiority trial was initiated. Design 2 considers the hypothetical scenario in which only the superiority RCT is run, as might have occurred if superiority had been expected from the start. Design 3 is a hybrid RCT-RWD study in which first a non-inferiority trial potentially augmented with RWD controls is run, and the follow-up superiority RCT is only initiated if the hybrid design does not reject the null hypothesis. This paper aims to compare Design 1 (which is currently in progress) to hybrid Design 3. Design 2 is also presented as a more traditional alternative to Design 1.

**Table 1.** *Causal Roadmap* steps for specification of study Designs 1–3

Roadmap Step	Designs 1–2 (RCT Only)	Design 3 (RCT + RWD)
1a. Causal question/ Causal estimand	What would the difference in risk of MACE^§^ (defined as death from any cause, nonfatal myocardial infarction, or nonfatal stroke) within one year be if all patients in a population consistent with the PIONEER 6 inclusion/exclusion criteria and timeframe [16], and with similar healthcare engagement, were prescribed oral semaglutide plus standard-of-care compared to if all patients were prescribed standard-of-care alone, and if censoring had been prevented for all patients? Note that the broad definition of the target population for all designs is patients meeting trial eligibility criteria and who might be likely to enroll in a trial based on their disease status and healthcare access and engagement. Although the baseline covariate distributions may differ between the RCT and RWD cohorts – leading to slightly different causal estimands – we do not aim to generalize results beyond the types of patients who would enroll in the RCT.	
	See Appendix 1 (Supplementary material 1) for the mathematical representation of the causal estimand for each study design.	
1b. Causal model	Knowledge about potential shared causes of treatment, censoring, MACE, and participation in the RCT vs RWD, as well as possible causal relations between these variables depicted in Fig. 2.	
2. Observed data	**Data sources used in this analysis:** Pioneer 6 RCT	**Data sources used in this analysis:** Pioneer 6 RCT, Optum CDM control arm,
	**Future data sources that would be used for the proposed designs:** SOUL RCT	
		**Future data sources that would be used for the proposed designs:** SOUL RCT
3. Assess identification	Identification highly likely (non-administrative censoring in PIONEER 6 only 0.3%)	Plausible, though uncertain, that causal gap^§§^ would be small (see Step 6).
4. Specify statistical estimand	Risk difference between treatment and control arms of the trial.	Adjusted risk difference between treatment and control arms, standardized to the covariate distribution in the target population.
5. Statistical model and estimator	Statistical Model: Semi-parametric statistical model (incorporating knowledge that treatment was randomized).	Statistical Model: Semi-parametric statistical model (incorporating knowledge that treatment in the RCT was randomized).
	Estimator: Unadjusted difference in risk between arms.	Estimator: Experiment-Selector CV-TMLE
6. Sensitivity analysis	None given that causal identification assumptions are highly likely to be true.	See Step 6 below.
7. Compare study designs	See simulation results reported in Step 7 below.	

CDM=Clinformatics® Data Mart Database; CV-TMLE=cross-validated targeted maximum likelihood estimator; MACE=major adverse cardiovascular events; RCT=randomized controlled trial; RWD=real-world data.

§ The revised definition of MACE using all-cause death instead of death from cardiovascular causes was chosen as the primary outcome because cause of death is not available in the RWD.

§§ The causal gap is the difference between the true value of the causal estimand that answers the causal question and the true value of the statistical estimand that we will estimate [9].

### Step 1a: Define the Causal Question and Estimand

The question for all study designs was what would the difference in risk of MACE (defined as death from any cause, nonfatal MI, or nonfatal stroke) within one year be if all patients in a target population consistent with the PIONEER 6 inclusion/exclusion criteria and timeframe [16], and with similar healthcare engagement, were prescribed oral semaglutide plus standard-of-care compared to if all patients were prescribed standard-of-care alone, and if censoring had been prevented for all patients? The outcome for this case study includes all-cause death (rather than cardiovascular death as in PIONEER 6) because the RWD does not include cause of death. The target population for all designs is a population of patients that not only meet the trial eligibility criteria but also are likely to enroll in a trial, based on their disease status and healthcare engagement. We do not aim to generalize the results of the trial to a new population. See Appendix 1 (Supplementary material 1) for the causal estimand.

### Step 1b: Specify a Causal Model

**Figure 2.**
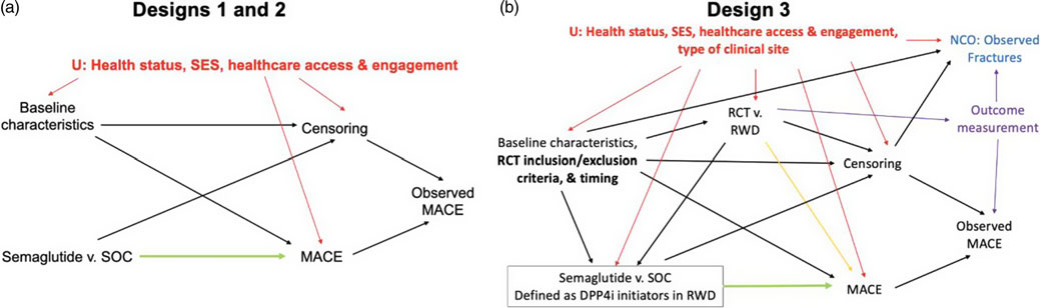
Causal graphs for Designs 1–3. DPP4i=dipeptidyl peptidase-4 inhibitor; MACE=major adverse cardiovascular events; NCO=negative control outcome; RCT=randomized controlled trial; RWD=real-world data; SOC=standard-of-care; SES=socioeconomic status.

Next, we specify a causal model for each design describing what variables might affect treatment, censoring, or outcomes using the causal graphs [25] shown in Fig. 2. For the RCTs, only the randomization procedure affects treatment assignment. As depicted in Fig. 2a, health status, socioeconomic status, and related issues of healthcare access and engagement, collectively referred to as *U*, might affect both censoring and MACE. Measured pre-baseline covariates, including age, sex, race, HbA1c, high-density lipoprotein cholesterol, low-density lipoprotein cholesterol, estimated glomerular filtration rate (a marker of kidney function), prior MI, prior stroke or transient ischemic attack (TIA), prior heart failure, morbid obesity, and use of glucose-lowering medications, insulin, and CV medications, may account for some aspects of these underlying factors.

In hybrid Design 3 (Fig. 2b), participation in the RCT versus the real-world system affects treatment because RCT participation is required to receive oral semaglutide if the RWD is concurrent with the pre-approval RCT. RWD controls receiving standard-of-care are selected based on initiation of a dipeptidyl peptidase-4 inhibitor (DPP4i), where conditioning on DPP4i initiation in the RWD is represented by the box around treatment in Fig. 2b. This choice is discussed in Step 3 and Appendix 2 (Supplementary material 1).

Being in the RCT could also modify the effect of treatment or directly affect measured outcomes for reasons including closer monitoring, encouragement of adherence, variation in standard-of-care or placebo effect, or more accurate outcome measurement [26–28]. The RCT inclusion and exclusion criteria, the timeframe of RCT recruitment, health status, socioeconomic status (SES), and healthcare engagement or access may also affect trial participation and MACE. Type of clinical site also affects trial participation – because the RCTs are conducted at research sites globally while the RWD comes from the United States and includes primary care practices – and may affect baseline characteristics, the definition of standard-of-care, censoring, and/or MACE.

Fig. 2b also includes fractures as a negative control outcome (NCO). NCOs are sometimes used to detect bias in observational studies [29,30]. An appropriate NCO is an outcome that is not affected by treatment but that is affected by the same sources of bias in the treatment effect estimate as the primary outcome. An observed association between the treatment and the NCO is then due either to finite sample variability or due to bias from sources including confounding, selection bias, measurement error, etc. [29,30]. From the available options, we chose fractures as an NCO because this outcome is generally serious enough to require medical attention for those with access, is associated with SES [31], and is recorded in a manner similar to the primary outcome. Fractures is not an ideal NCO, however; although closer monitoring in the RCT might improve cardiovascular outcomes, we do not expect closer monitoring to lead to decreased numbers of fractures (no yellow arrow from RCT v. RWD to the NCO in Fig. 2b). Studies prospectively designed to include an NCO could consider alternate choices to more comprehensively capture potential sources of bias.

### Step 2: Consider the Observed Data

Next, we consider the data that will be observed. Designs 1–2 propose to use data from one or both of the following RCTs: PIONEER 6 [16] and SOUL [17]. While PIONEER 6 has ended, the data from SOUL are not yet available. Both trials randomized participants to receive semaglutide or placebo plus standard-of-care. The inclusion and exclusion criteria for both trials targeted patients with T2DM and high cardiovascular risk but without unstable disease or recent use of a GLP-1RA, while PIONEER 6 also excluded recent users of pramlintide and DPP4is [17,19]. Participants were regularly evaluated in person or by phone. Outcomes were adjudicated. The timeframe for the outcome of one year after baseline was selected because no administrative censoring occurred before that time in PIONEER 6.

The external control arm considered in Design 3 could come from multiple sources, including the control arm of a previous trial such as SUSTAIN-6. While we would expect controls enrolled in a previous trial to be more similar to the current trial controls than participants in RWD, in this case study, we chose to consider RWD controls in order to discuss the many challenges that might arise in a hybrid study when a past trial’s control arm is not available. The RWD also has the advantage of being contemporaneous with the RCT, avoiding bias due to temporal differences between the RCT and external control arms. Specifically, the RWD considered in Design 3 came from Optum’s de-identified Clinformatics® Data Mart Database (CDM) (2007–2022), which is derived from administrative health claims for a geographically diverse population, spanning all 50 of the United States.

Consistent with recommendations from the RCT DUPLICATE study, we used naive initiation of a DPP4i (defined as a new prescription following at least 90 days without a previous prescription based on AHFS codes) to enhance comparability of healthcare access and engagement among RWD compared to RCT controls [32]. Time zero was defined as the first time a participant met the eligibility criteria for PIONEER 6, during a calendar time window contemporaneous with PIONEER 6 recruitment, and was prescribed a DPP4i.

We applied as many of the PIONEER 6 inclusion/exclusion criteria as possible given the available CDM data (Supplementary material 1, Appendix 7). Medical history variables that were part of these criteria or the baseline characteristics discussed in Step 1b were determined based on ICD-9/ICD-10 diagnosis and procedure codes. Medication use was identified through AHFS codes, where treatment at baseline corresponded to at least one prescription in the 180 days preceding time zero. To identify GLP1-RA and pramlintide usage for the exclusion criteria, we defined continuous treatment eras as consecutive prescriptions based on AHFS codes with no more than 90-day gaps between them. Laboratory measurements were identified through LOINC codes, where baseline values were identified as the most recent measurement within 180 days prior to time zero. For the primary and negative control outcomes, we identified nonfatal MI/stroke and fractures using ICD-9/ICD-10 codes for inpatient visits in the first diagnosis position (Supplementary material 1, Appendix 7). All-cause death was identified from external sources as provided by Optum.

### Steps 3-4: Assess Identifiability and Specify a Statistical Estimand

We now discuss whether the causal effect of interest (Step 1a) is identifiable from the observed data (Step 2). When using RCT data alone (Designs 1–2), we assume no unmeasured common causes of treatment or censoring and MACE and adequate data support (positivity) [33,34]. These assumptions are highly likely to hold by design; while it is possible that there are unmeasured common causes of censoring and MACE because censoring was negligible (0.3% in PIONEER 6), this would be unlikely to impact the results. An alternative design (distinct from Designs 1–3) in which we committed to augmenting the RCT data with external control data would require additional assumptions: (1) no unmeasured common causes of MACE and either selection into the RCT versus the RWD cohort, definition of standard-of-care in the RCT versus RWD, or censoring (no U in Fig. 2b), (2) no direct effect of RWD versus RCT participation on MACE or outcome measurement, and (3) adequate data support (positivity [33,34]).

In contrast, rather than rely on these assumptions holding, Design 3 estimates the magnitude of bias that would be introduced by RWD before deciding whether to integrate RWD with RCT data. If violation of these assumptions results in meaningful bias, Design 3 is likely to reject the RWD controls. In selecting candidate external controls for Design 3, the objective is to satisfy these assumptions to the extent possible, thereby increasing the probability that RWD will be integrated into the hybrid analysis, decreasing the probability that a follow-up superiority trial will be necessary, and minimizing risk of lower than nominal confidence interval coverage.

To improve the plausibility of identification assumptions, we selected RWD controls with a similar disease stage and healthcare engagement compared to the RCT controls based on those who were prescribed an active comparator medication (DPP4i) and had relevant baseline labs and medical history recorded. The aim was to minimize the impact of factors like health status and healthcare engagement on selection into the RCT versus the external cohort and on the definition of standard of care in the two studies. Yet this step also restricts the target population for the hybrid analysis to patients similar to those who actually enrolled in the RCT. Because DPP4i use was an exclusion criterion in PIONEER 6, we also require the assumption, supported by data, that DPP4is do not influence cardiovascular outcomes [32]. We also restricted RWD controls to the time period of PIONEER 6 recruitment to make standard-of-care more similar, and we selected RWD controls whose baseline characteristics were shared by at least some RCT participants. This last step was necessary to avoid a violation of the positivity assumption and further restricted the target population.

As is generally true when observational data are considered, it remained unlikely that all causal identification assumptions held completely. Yet with the above considerations, it was plausible that the bias from integrating RWD would be small. Design 3 evaluates this claim empirically. Appendix 1 (Supplementary material 1) provides statistical estimands that are equivalent to the treatment effects of interest, or causal estimands, under identification assumptions. Appendix 2 (Supplementary Material 1) provides further discussion of these assumptions.

### Step 5: Choose a Statistical Model and Estimator

Next, we chose a statistical estimator. In Designs 1–2, because censoring was negligible, we estimated the unadjusted risk difference between arms among persons with follow-up through one year. For Design 3, we used an estimator designed to analyze hybrid studies.

Multiple hybrid-design estimators have been developed in recent years, with the common goal of evaluating whether to include (or how to weight) external data in a hybrid analysis based on the difference in mean outcomes, conditional mean outcomes, or effect estimates estimated using external data or estimated using RCT data alone. These estimators differ based on whether they take a Bayesian [35–38] or Frequentist [5,6,24,39,40] approach, their criteria for deciding whether to include or how to weight external data, the estimator used for effect estimation, and the method of confidence interval construction. We refer the interested reader to Lim *et al*. (2018) [4] and Oberst *et al*. (2022) [40] for more detailed explanations of alternative methods.

In this case study, we use the experiment-selector cross-validated targeted maximum likelihood estimator (ES-CVTMLE) [5] as the estimator for Design 3. Cross-validated Targeted Maximum Likelihood Estimation (cv-TMLE) is a robust, efficient approach that incorporates machine learning using cross-validation [7,41–43]. This allows it to flexibly adjust for covariates without introducing new assumptions, improving precision and potentially reducing bias while preserving inference [7,41–43]. Censoring is handled by incorporating inverse probability of censoring weights into the TMLE targeting procedure [44].

The ES-CVTMLE extends the cv-TMLE method to evaluate and integrate external data [5]. First, the ES-CVTMLE estimates the bias introduced by augmenting the RCT with RWD by comparing the conditional mean outcomes for RCT and RWD controls (see Supplementary material 1, Appendix 3) [5]. ES-CVTMLE also estimates bias as the estimated average treatment effect on the NCO. Dang *et al*. (2022) describe how these two bias estimates are used to decide whether to integrate RWD or to analyze the RCT data alone [5].

In this case study, we chose the ES-CVTMLE for Design 3 because it relies on few statistical assumptions, incorporates an estimate of bias based on an NCO, and adjusts confidence interval widths based on the estimated magnitude of bias. While the focus of this case study is on using simulations to compare study designs, similar simulations could also be used to compare different potential estimators. Appendices 3–6 provide further details about estimation.

### Step 6: Specify a Procedure for Sensitivity Analysis

*Causal Roadmap* Step 6 is to conduct a sensitivity analysis to evaluate how violations of identification assumptions might impact conclusions. Because the topic of sensitivity analysis is complex, we do not cover this step in this case study but instead refer readers to the companion paper that reviews sensitivity analysis in detail.

### Step 7: Compare Study Designs Using Simulations

We compare Designs 1–3 using simulations that mimic our true study designs (Supplementary Material 1, Appendix 4). The code to run these simulations may be found in Supplementary Material 2. For Designs 1 and 3, we simulate data from a small RCT aiming to mimic PIONEER 6 (RCT1). For Design 1, we use an unadjusted estimate of the difference in risk between arms of RCT1. For Design 3, we consider simulated RCT1 and a simulated “real-world” dataset aiming to mimic CDM, and we use the ES-CVTMLE to estimate the risk difference. For the sake of comparison to a more standard estimator, we also report the results of simulated Design 3 if a cv-TMLE with no assessment of bias had been used to analyze the pooled RCT and RWD. In both Designs 1 and 3, if the null hypothesis is rejected at this first stage, we use this initial estimate as our final effect estimate. If the null hypothesis is not rejected, then we simulate data from a larger trial aiming to mimic a superiority trial (RCT2) and estimate the risk difference using an unadjusted estimate. In Design 2, we use the unadjusted effect estimate from RCT2.

Because Design 3 considers non-randomized data, it is possible that confidence interval coverage will be lower than in Designs 1–2 if both the causal identification assumptions (Step 3) are violated and the empirical bias estimate based on the available finite sample is smaller than the true bias (leading to inappropriate inclusion of external controls). Whether this risk is acceptable will depend on the context, including the magnitude of benefit to patients of Design 3 over Designs 1–2. While there are many potential ways to quantify benefit to patients, here we estimate the number of patient-years during which participants are precluded from starting a GLP1-RA (by being in an RCT control arm), averaged over 1000 iterations of this simulation. We also report power to detect the true effect using α = 0.05. Because participation in an RCT requires acceptance of potential risks and commitment of time to the study, it is important that any design that involves an RCT be adequately powered to answer the question of interest [45].

We evaluate Design 3 when the magnitude of bias introduced by including simulated RWD is zero and when it is one of ten potential magnitudes in either direction up to ± 2.1%. In this primary simulation, the effect of unmeasured factors causing bias is the same on the relationship between semaglutide and MACE as it is on the relationship between semaglutide and the NCO. Appendix 5 (Supplementary material 1) shows the results of the same simulation both when the NCO is not considered and when the NCO is considered but the unmeasured factors causing bias for the relationship between semaglutide and MACE have no effect on the NCO, mimicking a worst-case scenario for violations of both the causal identification assumptions and the assumptions needed to measure bias using an NCO.

## Results

### Simulation Results

**Figure 3.**
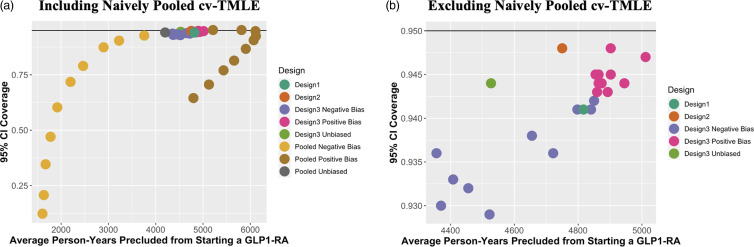
Simulation results by study design with different amounts of RWD bias. The “Pooled” analysis is Design 3 where the pooled simulated RCT1 and RWD are analyzed with a standard cv-TMLE. Purple and gold represent 10 simulated magnitudes of bias away from the null for the ES-CVTMLE and naively pooled cv-TMLE estimators. Pink and brown represent 10 simulated magnitudes of bias toward the null for the ES-CVTMLE and naively pooled cv-TMLE estimators. CI=confidence interval; GLP1-RA=glucagon-like peptide-1 receptor agonist; cv-TMLE=cross-validated targeted maximum likelihood estimator; ES-CVTMLE=experiment-selector cv-TMLE; RWD=real-world data.

Fig. 3 shows the results of 1000 iterations of the simulation comparing Designs 1–3. Designs 1 and 2 had similar characteristics. Simulated Design 1 had 95% CI coverage of 0.941, power of 0.835, and an average of 4,817 patient-years during which a GLP1-RA was precluded. Simulated Design 2 had coverage of 0.948, power of 0.775, and an average of 4,750 patient-years during which a GLP1-RA was precluded. Below, we focus on the differences between Design 1 (which is currently in progress) and Design 3 (the hybrid trial).

The tradeoffs between Design 3 and Design 1 depended on the direction of bias introduced by the RWD. With unbiased simulated RWD, Design 3 had coverage of 0.944, had power of 0.854, and resulted in an average of 290 fewer participant-years during which patients were precluded from starting a GLP1-RA compared to Design 1. In other words, on average, 6% fewer people would have spent one year during which their doctor avoided prescribing a GLP1-RA if Design 3 were chosen and unbiased RWD were available compared to if Design 1 were chosen. If simulated RCT1 and RWD had been pooled and analyzed with a cv-TMLE instead, Design 3 with unbiased RWD would have had coverage of 0.941, power of 0.862, and an average of 618 fewer participant-years during which GLP1-RA use was discouraged. However, the downsides of this approach, which relies on causal identification assumptions rather than evaluating them empirically, are apparent (Fig. 3a) when biased simulated RWD is considered.

Positive bias (toward the null) represents scenarios in which the introduction of RWD lowers the estimated risk of MACE among control arm participants. This could happen if MACE were not well-recorded in the RWD. For Design 3, simulated positive bias led to coverage ranging from 0.943 to 0.948, power ranging from 0.825 to 0.834, and an average of 38 to 195 extra participant-years during which prescription of a GLP1-RA was discouraged compared to Design 1. The increase in person-years without GLP1-RA access occurred because RWD with bias toward the null was included in a small number of simulation iterations, triggering a second RCT. As shown in Fig. 3a, the increase in person-years without GLP1-RA access would have been much larger if a naïve pooled analysis had been conducted for Design 3.

Negative bias (away from the null) represents scenarios in which the introduction of RWD raises the estimated risk of MACE among control arm participants. This could happen if the subjects whose data were collected in the RWD showed more severe health outcomes (conditional on measured factors that affect health outcomes) than the trial participants due to differences described in Step 1b. Simulated negative bias led to coverage for Design 3 ranging from 0.929 to 0.942, power ranging from 0.834 to 0.860, and an average of 33 more to 461 fewer participant-years during which prescription of a GLP1-RA was discouraged compared to Design 1. For comparison, if one had used a naïve cv-TMLE estimator to analyze the pooled simulated RCT1 and RWD, coverage would have been as low as 0.123 (Fig. 3a). The ES-CVTMLE thus provided significant (though imperfect) protection against integration of biased RWD in this simulation. The possibility of bias away from the null is plausible. Nonetheless, by objectively quantifying these differences between proposed designs, investigators can explicitly discuss these tradeoffs with stakeholders such as patient groups and regulatory agencies when selecting a trial design.

### Real Data Analysis

The actual results of Designs 1 and 2 await completion of the SOUL trial. Below, we carry out Design 3 using data from PIONEER 6 and the CDM external control arm described in Step 2. We also report the results of an unadjusted estimator for the difference in the risk of MACE among PIONEER 6 active and control arm participants.

**Figure 4.**
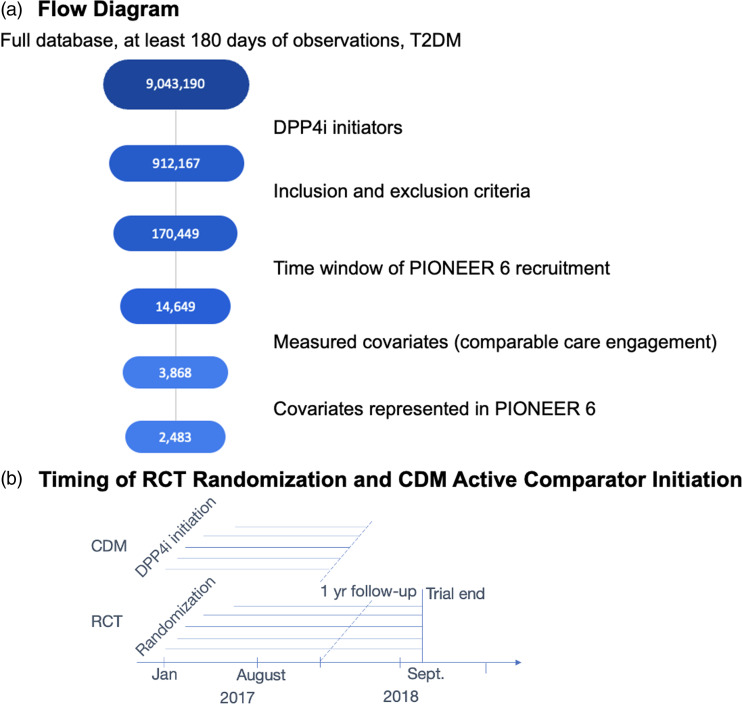
Selection of CDM external control group. CDM=Clinformatics® Data Mart Database; T2DM=type 2 diabetes mellitus; DPP4i=dipeptidyl peptidase-4 inhibitor; Jan=January; RCT=randomized controlled trial; Sept=September; yr=year.

After applying the inclusion and exclusion criteria described in Step 2 and depicted in Fig. 4, the CDM cohort consisted of 2483 participants. Table 2 lists baseline demographics, medical history, medication use, outcome missingness, and MACE and NCO event rates for the PIONEER 6 semaglutide and placebo arms, as well as for the CDM external control arm. The outcome was missing for 0.3% of PIONEER 6 participants and 16% of CDM participants. Compared to the PIONEER 6 control arm, the CDM controls were slightly older, had a higher percentage of females, had a lower proportion of previous MI or stroke but a higher proportion of previous heart failure, and had a different distribution of baseline medication use. These differences in baseline covariates reflect differences in the clinical settings and patient populations represented by the RCT and RWD that are adjusted for in the analysis.

**Table 2.** Baseline characteristics, outcome missingness, and event rates for PIONEER 6 and CDM

	CDM RWD control arm (*n* = 2483)	PIONEER 6 placebo arm (*n* = 1564)	PIONEER 6 semaglutide arm (*n* = 1574)
MACE rate - %	4.5	4.2	2.9
NCO Rate - %	0.73	0.77	0.45
Age - years, mean (SD)	69.2 (6.3)	66.4 (7.1)	65.9 (7.1)
Female sex - %	42.7	31.2	31.9
Race			
White - %	44	72	72
Black - %	11	7	6
Other - %	45	21	22
HbA1c - %, mean (SD)	8.0 (1.4)	8.2 (1.6)	8.2 (1.6)
LDL cholesterol - mg/dl, mean (SD)	84.1 (28.3)	84.8 (32.4)	83.9 (34.0)
HDL cholesterol - mg/dl, mean (SD)	44.1 (9.6)	41.6 (10.7)	41.9 (11.0)
eGFR - ml/min/1.73 m2, mean (SD)	74.3 (19.4)	74.2 (20.9)	74.2 (21.1)
Previous MI - %	13.9	36.9	35.3
Previous stroke/TIA - %	11.8	16.6	15.2
Previous heart failure - %	20.4	12.4	11.9
Morbid obesity - %	16.4	12.3	12.2
Glucose-lowering medication (metformin, SU, TZD, SGLT2i)	73.0	83.9	83.9
Insulin	14.9	61.2	61.2
Cardiovascular medication (antihypertensives, lipid-lowering, anti-thrombosis, diuretics)	91.5	98.9	98.9
Outcome missingness - %	15.8	0.3	0.3
NCO missingness -%	17.2	0.3	0.3

CDM=Clinformatics® Data Mart Database; eGFR=estimated glomerular filtration rate; HbA1c=glycated hemoglobin; HDL=high-density lipoprotein; LDL=low-density lipoprotein; RWD=real-world data; MACE=major adverse cardiovascular events; MI=myocardial infarction; mg/dl=milligrams per deciliter; ml/min/1.73 m2=milliliters per minute per 1.73 square meters of body surface; NCO=negative control outcome; SD=standard deviation; SGLT2i=sodium/glucose cotransporter-2 inhibitor; SU=sulfonylurea; TIA=transient ischemic attack; TZD=thiazolidinedione.

**Figure 5.**
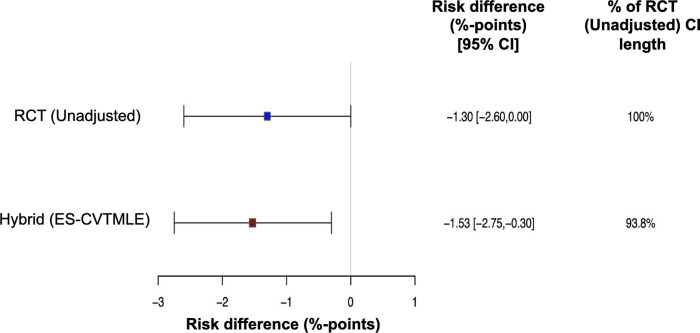
Estimated difference in 1-year risk of MACE for PIONEER 6 and hybrid design. CI=confidence interval; ES-CVTMLE=experiment-selector cross-validated targeted maximum likelihood estimator; MACE=major adverse cardiovascular events; RCT=randomized controlled trial.

As shown in Fig. 5, the estimated difference in the risk of MACE by 1 year based on the unadjusted estimator conducted using PIONEER 6 data was –1.30%-points (95% CI –2.60%–0.00%-points). This result is closer to statistical significance than the primary result reported for the PIONEER 6 trial (HR 0.79; 95% CI 0.57–1.11 [16]) because the primary analysis (a) evaluated the hazard ratio including all timepoints instead of the risk difference by one year and (b) evaluated a composite outcome that included death from cardiovascular causes instead of death from all causes.

Hybrid Design 3 resulted in an estimated risk difference of –1.53%-points (95% CI –2.75– –0.30%-points), providing evidence in support of the superiority of oral semaglutide versus standard-of-care for the prevention of MACE. The two primary differences between the risk difference estimates from PIONEER 6 alone compared to the hybrid analysis are narrower confidence intervals and a small negative shift in the point estimate. Narrower confidence intervals resulted from the data-adaptive estimator accepting the CDM RWD for inclusion in the analysis in 84% of cross-validation splits, leading to increased efficiency.

The small shift in the point estimate may have occurred for three main reasons. First, the magnitude of the shift is well within what might be expected by chance alone. Second, the shift may be due to subtle changes in the target population that arise from including external controls despite using the same eligibility criteria. Finally, the potential for some residual bias remains.

## Discussion

In this case study, we demonstrate an application of the *Causal Roadmap* to a hybrid randomized RWD trial. We discuss considerations for improving the plausibility of identification assumptions in this data fusion context. We also implement the extension proposed in the companion article [10] to use the *Causal Roadmap* to compare different potential study designs using simulations.

Both the FDA guidance on complex innovative trial designs [46] and the FDA guidance on adaptive designs [47] suggest the utility of simulations for comparing alternative design choices. In this case study, the simulation demonstrates how sponsors may quantify the tradeoffs between different study designs. Compared to Design 1, Design 3 with unbiased external data led to similar confidence interval coverage but less time during which patients were precluded from receiving a GLP1-RA. This suggests that Design 3 would be superior if unbiased external data were available.

Yet with simulated bias toward the null, Design 3 had similar coverage but potentially more patient time without a GLP1-RA compared to Design 1, defeating the purpose of the hybrid design. It would thus be important to evaluate how likely this scenario would be before proposing Design 3. For example, a study could be conducted to compare outcomes recorded in CDM to outcomes recorded following an RCT protocol to assess the likelihood that bias toward the null would occur due to under-reporting of outcomes in the RWD.

Compared to Design 1, Design 3 with bias away from the null generally resulted in less time during which a GLP1-RA was avoided, but coverage fell below 95% for intermediate magnitudes of bias. These results are consistent with simulations and theoretical assessments of other estimators for hybrid randomized-external data designs that have demonstrated that hybrid designs cannot both (1) improve power or mean squared error (MSE) compared to an RCT alone; and (2) guarantee 95% confidence interval coverage (or no decrease in MSE) regardless of the magnitude of bias introduced by the external data [5,40]. The risk of below-nominal coverage with certain magnitudes of bias must be weighed against the benefit of allowing more patients to start a GLP1-RA earlier. We do not advocate for one design over another in this case study but rather aim to demonstrate how tradeoffs can be quantified to facilitate discussion with regulatory agencies and patient groups.

Our estimate of the difference in the risk of MACE by one year with oral semaglutide versus standard-of-care supports the superiority of oral semaglutide, but regulatory decisions regarding whether to extend the label of oral semaglutide to include the secondary indication of cardiovascular risk reduction will await the results of the SOUL trial. A key limitation of this study is that it was planned after PIONEER 6. RWD studies aiming to support policy and/or regulatory decision-making must pre-specify all design and analysis decisions before effects are estimated from any of the proposed data sources [10]. The *Causal Roadmap* supports a rigorous design process and reporting structure to ensure pre-specification of components needed to support the validity of causal inferences drawn from such designs. The current case study provides a detailed work example of this process.

While this study aimed to quantify tradeoffs between three proposed designs, other approaches could be considered. Hybrid RCT-RWD designs may adapt the probability of randomization to active treatment based on the efficiency gains that are achieved by integrating RWD and also on the probability of superiority compared to placebo [36,48,49], potentially leading to even less patient time on an inferior product. Power could also have been higher if oral semaglutide had been available in the CDM dataset for the specified time period – resulting in integration of both extra treatment and control arm participants – or if more RWD controls were available.

This study reports one of many potential metrics aimed at quantifying the benefits and drawbacks of different study designs from the perspective of patients. While recommendations have been proposed regarding the elicitation of patient perspectives to inform medical product development [50,51], further guidance on the most relevant metrics of patient benefit as well as best practices for collaboratively weighing tradeoffs between different metrics of design performance is warranted. Such guidance would help to inform future applications of the *Causal Roadmap* for pre-specification of hybrid randomized-RWD designs.

## Supporting information

Dang et al. supplementary material Dang et al. supplementary material

## Supplementary material

The supplementary material for this article can be found at https://doi.org/10.1017/cts.2023.656.
