# Supplementary material for: Case study of semaglutide and cardiovascular outcomes: An application of the Causal Roadmap to a hybrid design for augmenting an RCT control arm with real-world data
Source: J Clin Transl Sci. 2023 Oct 23;7(1):e231. doi: 10.1017/cts.2023.656 (PMC10643919; doi:10.1017/cts.2023.656)
Supplement: Dang et al. supplementary material [file S2059866123006568sup001.docx]

**Supplementary Appendices**

**Appendix 1: Mathematical Notation for Causal and Statistical Estimands**

As described in Table 1, the causal question of interest is: what would the difference in risk of MACE (defined as death from any cause, nonfatal MI, or nonfatal stroke) within one year be if all patients in a population consistent with the PIONEER 6 inclusion/exclusion criteria and timeframe [1], and with similar healthcare engagement, were prescribed oral semaglutide plus standard-of-care compared to if all patients were prescribed standard-of-care alone, and if censoring had been prevented for all patients?

The two intervention variables that are modified in our treatment strategies are *A* – an indicator of prescribing patients oral semaglutide in addition to standard-of-care (*A=1*) or standard-of-care (*A=0*) – and *C* – an indicator of whether the participants were censored before one year. We denote our outcome of MACE by 1-year as *Y*. Because some participants are censored, the observed outcome, $Y^{\star}$, is the true value of MACE for those who were not censored and whose outcomes were measured correctly and missing for those who were censored.

We then define the following potential outcomes [2,3]; $Y^{a=1,c=0}$ is the one-year MACE status an individual would have had if they had been prescribed oral semaglutide in addition to standard-of-care and not been censored, and $Y^{a=0,c=0}$ is the one-year MACE status an individual would have had if they had been prescribed standard-of-care and not been censored. The simplest mathematical representation of a causal estimand that answers our question is given by the causal risk difference:

$$E\left( Y^{a=1,c=0} - Y^{a=0,c=0} \right).$$

Note that this causal risk difference is defined with respect to a specific target population. Despite efforts to ensure comparability between the RCT population and external control RWD, our approach acknowledges that the RCT and RWD populations may nonetheless have different distributions of baseline characteristics. Because the proposed estimator (ES-CVTMLE) only augments the control arm when the RWD meet pre-specified criteria (evaluated across multiple internal sample splits), the exact target population to which the causal risk difference applies will depend on the extent to which these criteria are met and the RCT standard-of-care arm is augmented with RWD.

More formally, let *S* be a variable describing study participation, where *S=0* indicates that an individual participated in an RCT and *S=1* indicates that an individual participated in the real-world healthcare system. **Designs 1 and 2** only utilize RCT data, and so in these designs, we can only evaluate the causal risk difference within the RCT context and for a target population represented by the RCT participants. We can rewrite the causal parameter to represent the causal risk difference (not adjusted for baseline characteristics) in a way that makes explicit that it refers to the RCT context:

$$\psi_{RCT,unadj}^{*}= {E(Y^{a=1,c=0} - Y^{a=0,c=0}|S=0)}_{.}$$

With baseline covariates, *W*, the adjusted causal risk difference for the RCT context and target population is:

$$\psi_{RCT,adj}^{*}= E_{W|S=0}[{E(Y^{a=1,c=0} - Y^{a=0,c=0}|W,S=0)]}_{.}$$

We note that the true causal risk difference in the non-inferiority and superiority trials could be different if they had different inclusion and exclusion criteria or if there were changes over time in the background standard-of-care. For simplicity, however, we will consider that the non-inferiority and superiority RCTs target the same causal parameter, $\psi_{RCT,unadj}^{*}$.

In hybrid **Design 3,** we consider integrating extra RWD controls with our non-inferiority trial and only run the superiority RCT if the null hypothesis of the superiority RCT is not rejected in the hybrid analysis. The ES-CVTMLE adjusts for baseline covariates whether the RWD is included or rejected, so if the hybrid design rejects the RWD, analyzing the non-inferiority RCT only, then the causal target parameter is $\psi_{RCT,adj}^{*}$.

In contrast, if the hybrid design does select to augment the non-inferiority trial with extra RWD controls, this may modify the target population if the RWD controls have a different (though overlapping) distribution of baseline covariates compared to the RCT population. Inclusion of RWD controls may also modify the target parameter if the true effect of oral semaglutide versus standard-of-care is different in the RCT and RWD contexts. The causal risk difference in the combined RCT plus RWD experiment that integrates *S=0* and *S=1* is given by:

$\psi_{RCT,RWD}^{*}= E_{W|S\in\{0,1\}}[E(Y^{a=1,c=0} - Y^{a=0,c=0}|W,S\in\{0,1\})]$.

In the hybrid design, we use the data from the real-world source and the non-inferiority trial to decide whether to estimate the causal risk difference for the RCT context and target population ($\psi_{RCT,adj}^{*}$) or the causal risk difference for the hybrid RCT-RWD context and target population ($\psi_{RCT,RWD}^{*}$), where either parameter represents an answer to our question for that particular population and context.

We will ultimately use the experiment-selector CV-TMLE [4] to analyze the results of the hybrid trial. This method uses cross-validation to separate the part of the data that is used to choose whether to attempt to estimate $\psi_{RCT,adj}^{*}$ or $\psi_{RCT,RWD}^{*}$ and the part of the data that is used for estimation of the corresponding risk difference. The decision of whether to augment the RCT with external control data may differ in different cross-validation folds. The causal estimand would then be interpreted as the causal risk difference for a target population that is a weighted average of the RCT population and external control RWD population. More formally, let the target parameter chosen for a given fold, *v*, be $\psi_{v}^{*}$. The overall causal target parameter for the hybrid design is then the average of the causal target parameters selected in each fold. For example, with ten cross-validation folds, the causal target parameter would be

$$\psi_{hybrid}^{*} = \frac{1}{10}\sum_{v=1}^{10} \psi_{v.}^{*}$$

Please see Dang et al. (2022) [4] for further details of this methodology.

Using the g-formula [5], we may define the statistical estimands (functions of the observed data) that are as close as possible to the causal effects of interest for each study design, where

$$\psi_{RCT,unadj}=E\left( Y^{\star} | C=0,A=1,S=0 \right)-E\left( Y^{\star} | C=0,A=0,S=0 \right),$$

$$\psi_{RCT,adj}= E_{W|S=0}\left[ E\left( Y^{\star} | C=0,A=1,W,S=0 \right)-E\left( Y^{\star} | C=0,A=0,W,S=0 \right) \right],$$

$$\psi_{RCT,RWD}= E_{W|S\in\{0,1\}}\left[ E\left( Y^{\star} | C=0,A=1,W,S\in\left\{ 0,1 \right\} \right)-E\left( Y^{\star} | C=0,A=0,W,S\in\left\{ 0,1 \right\} \right) \right],$$

$\psi_{v}$ is whichever of $\psi_{RCT,adj}$ or$\psi_{RCT,RWD}$ was selected in cross-validation fold *v*, and

$$\psi_{hybrid} = \frac{1}{10}\sum_{v=1}^{10} \psi_{v}.$$

**Appendix 2: Assessment of Plausibility of Causal Identification Assumptions**

First, we consider whether the causal effect is identified in **Designs 1 and 2** based on the directed acyclic graph (DAG) in Figure 2a, using the backdoor criterion [6]. Because treatment was randomized, there are no unmeasured common causes of treatment and the outcome. Furthermore, because censoring is minimal in the RCTs (it was truly 0.3% by one year in PIONEER 6) [1], the magnitude of bias that could result from potential unmeasured common causes of censoring and MACE is likely to be negligible. For these reasons, we expect to identify the causal effect of interest using **Designs 1 and 2**.

As discussed in the main text, **Design 3** does not assume that the causal effect of interest is identified in the pooled RCT and RWD. However, taking steps to improve the plausibility of causal identification assumptions for the combined data also increases the likelihood that RWD will be integrated in the hybrid design. Again using the backdoor criterion [6] and our DAG in Figure 2b, we consider possible reasons for a causal gap in an analysis of the pooled RCT and RWD. First, there would be a causal gap if being in the RCT versus the real world affected outcomes outside of the effect due to prescribing either oral semaglutide or standard-of-care; this is certainly possible for the reasons described in Step 2 above.

If we were able to conduct a pragmatic clinical trial for the randomized component of our hybrid design in which the trial aimed to mimic real-world care as closely as possible outside of baseline treatment randomization, then it would be more likely that trial participation only affected outcomes through treatment assignment [7,8]. In this case, however, we are not able to consider a pragmatic RCT because the current FDA draft guidance on “Evaluating the Safety of New Drugs for Improving Glycemic Control” [9] requires a sufficient number of phase 3 clinical trial patient-years on the medication of interest during which time CV outcomes are evaluated by adjudication to evaluate cardiovascular safety. Instead, we may attempt to select RWD controls who at least have similar healthcare access and engagement compared to RCT participants (discussed below).

There would also be a causal gap for the pooled RCT and RWD analysis if there were unmeasured common causes of trial participation and MACE or of censoring and MACE, and we expect a larger amount of censoring in the real world compared to the RCT. We apply the same inclusion and exclusion criteria and timeframe for the RWD and RCT controls, yet the question remains whether the measured baseline characteristics that are indicative of demographics, baseline health status, and treatment are sufficient to adjust for common causes of our intervention variables and outcome.

To try to minimize the amount of bias that would be introduced by integrating RCT and RWD controls, we consider a further restriction of the CDM cohort to select patients who are likely to be at a similar disease stage with similar healthcare access and engagement compared to the RCT participants. Selecting RWD patients prescribed DPP4is is one method of making disease stage and engagement comparable [10]. Although Chiu and Dahabreh (2022) note that conditioning on specific levels of treatment may introduce collider bias by creating an association between trial participation and the outcome if there were no unmeasured common causes of trial participation and the outcome prior to conditioning on treatment [11], in this case, we think that the unmeasured factors in Figure 2b (health status, socioeconomic status, healthcare access and engagement, and type of clinical site) would have a larger impact on inclusion in the RCT versus the external control arm and on the definition of standard-of-care in the RCT versus the external data if no active comparator had been used in the RWD. We also exclude CDM patients with missingness in the baseline covariates, expecting that patients for whom this laboratory and medical history data is not recorded might not be followed as closely by their providers as patients in the RCT.

Additionally, there would be a causal gap if assignment to standard-of-care in the trial and the RWD were not equivalent in terms of their effects on MACE. The most obvious reason this might not be true is that participants in the RCT were prescribed an inactive placebo pill and were not prescribed a DPP4i based on exclusion criteria, while the RWD participants were prescribed a DPP4i as an active comparator. The question then is whether the effect of being assigned placebo is different from the effect of being prescribed a DPP4i on the outcome of MACE. In the RCT Duplicate study, DPP4is were chosen as a “proxy for placebo” relative to the outcome of MACE in studies of GLP-1RAs “because they are antidiabetic treatments that have similar indications to the treatments under study, but they are not known to have any effect on the cardiovascular outcomes of interest based on recent evidence” [10]. If this reasoning is correct, assignment to placebo should have the same effect as assignment to a DPP4i for the primary outcome. Another question is whether the background standard-of-care that patients receive is equivalent in the RCT and the RWD. While it is possible that there are differences between the standard-of-care provided by trial versus non-trial clinicians, we attempted to ensure that “standard-of-care” would be as similar as possible by restricting the CDM cohort to the same time period as PIONEER 6.

Finally, to identify a causal effect in the combined RCT and RWD, we need sufficient data support. In other words, participants in any stratum of measured confounders must have a positive probability of being assigned to either intervention strategy: oral semaglutide and not being censored or standard-of-care and not being censored. This assumption is also known as the positivity assumption [12,13]. Because we only add extra RWD controls, including any RWD participant whose particular combination of measured potential confounding variables was not shared by RCT participants would violate the positivity assumption. We solve this problem by limiting the CDM cohort to participants whose baseline covariates were within the range of baseline covariates represented in the trial population.

**Appendix 3: Estimation of the Causal Gap**

The first estimate of the causal gap used by the ES-CVTMLE compares conditional mean outcomes between RCT and combined RCT-RWD controls. The statistical estimand for this causal gap parameter is given by

$\Psi^{\#}= E_{W|S\in\{0,1\}}\left[ E\left[ Y^{\star} | C=0,A=0,S=0,W \right]-E\left[ Y^{\star} | C=0,A=0,S \in\left\{ 0,1 \right\},W \right] \right].$

The ES-CVTMLE estimates $\Psi^{\#}$ using targeted maximum likelihood estimation [14,15], but the precision of the estimate depends on the sample size of the RCT. In a given sample dataset, the estimate of $\Psi^{\#}$ will not be exactly equal to the true causal gap because of finite sample variability. Nonetheless, $\Psi^{\#}$ represents our best estimate of the causal bias that would be introduced by including RWD controls in the analysis. See Dang et al. (2022) [4] for more details.

We also estimate the causal gap as the estimated average treatment effect on a negative control outcome (NCO). NCOs are not affected by the treatment but ideally should be affected by as many of the factors that lead to violations of identification assumptions as possible [16]. Any estimated association between treatment and the NCO is thus due either to a causal gap or due to finite sample variability.

**Appendix 4: Simulation set-up**

The data for the simulation were generated as follows. First, we generate data to mimic a non-inferiority RCT (RCT1) of sample size *n_1_*=3183, twenty-one different “real-world” datasets (RWD) of sample size *n_2_*=2483, and a superiority RCT (RCT2) of sample size *n_3_*=9500. In the two “RCT” datasets, treatment is randomized with probability 0.5. In the “RWD”, all participants receive *A=0*. Two baseline covariates, *W1* and *W2* are drawn from $Normal(\mu=0,\sigma=1)$ distributions for participants from all studies.

We generate 21 potential levels of bias in the “RWD” as follows. *B* is a variable that introduces bias when non-zero. The value of *B* is zero for the two “RCT” datasets and the unbiased “RWD” dataset. For the remaining 20 “RWD” datasets, the value of *B* ranges from positive $\frac{1}{10}*0.65$ to $\frac{10}{10}*0.65$ in increments of $\frac{1}{10}*0.65$ and from $\frac{-1}{10}*1.7$ to $\frac{-10}{10}*1.7$ in increments of $\frac{-1}{10}*1.7$. These values were chosen because, due to the properties of the ${logit}^{-1}$ function, this range of values of *B* leads to true bias as large as ±2.1%. This maximum magnitude of bias in either direction was chosen so that the true bias minus two times the standard error of the bias estimator would be larger than the standard error of the risk difference TMLE estimator for the RCT alone. Because the ES-CVTMLE will select the combination of RCT and RWD if the estimated squared bias plus the variance of the TMLE risk difference estimator for the combined data is smaller than the estimated squared bias plus the variance of the TMLE risk difference estimator for the RCT alone, these magnitudes of bias include the full spectrum of magnitudes for which we would expect that RWD might be included in the analysis in some simulation iterations.

The primary outcome, *Y*, is generated as follows:

$$Y \sim Bernoulli(p ={logit}^{-1}\left( -3.33 + 0.2*W1 - 0.4*W2 + U_{y}+B \right))$$

where $U_{y} \sim Normal(\mu=0,\sigma=0.5)$. This equation was designed so that the overall probability of MACE would be similar to the true probability of MACE in the PIONEER 6 placebo arm (4.2%). Adding extra random error, $U_{y}$, means that the baseline covariates are not very predictive of the outcome, which is common with relatively rare binary outcomes measured years after baseline.

In order that the magnitude of the effect of *B* on the relationship between the treatment and the negative control outcome (NCO) be similar to the magnitude of the effect of *B* on the relationship between the treatment and the true outcome, but to make sure that the primary and negative control outcomes are not too tightly correlated, we let

$$NCO \sim Bernoulli(p ={logit}^{-1}\left( -3.33 + 0.2*W1 - 0.4*W2 + U_{nco} +B \right))$$

where $U_{nco} \sim Normal(\mu=0,\sigma=0.5)$ but is independent of $U_{y}.$ The simulation in Appendix 5 describes an alternate, “worst-case” simulation in which *B* has no effect on the NCO.

We also generate some missing outcomes, where the indicator that the outcome is censored ($C=1$) and the indicator that the NCO is censored ($C_{nco}=1$) are generated as follows:

$$C \sim Bernoulli(p =\left( 1-{logit}^{-1}\left( 2.2 + W1 - W2 + 4.5*I\left( S=0 \right) \right) \right))$$

$$C_{nco} \sim Bernoulli(p =\left( 1-{logit}^{-1}\left( 2.2 + W1 - W2 + 4.5*I\left( S=0 \right) \right) \right))$$

where *S=0* indicates one of the simulated RCT datasets. These equations for outcome missingness were designed to approximate the true rates of outcome missingness in the RCT context (0.3% for PIONEER 6) and the RWD context (16% for CDM).

Note that in this simulation, treatment, *A,* does not affect the outcome, *Y*. If treatment were to affect the outcome, then the true causal risk difference (CRD) would be different for different values of *B*, even if there were no interaction term between *B* and *A*, due to the properties of the ${logit}^{-1}$ function. Instead, because *A* does not affect *Y*, the true value of the CRD for all combinations of RCT and RWD is zero. This sets up an even competition between the study designs when different RWD are included in the hybrid analysis; we would expect the power to reject the same null hypothesis to depend on bias and variance but not on different true causal effects when different combinations of data are analyzed. We report 95% CI coverage for the true causal risk difference of zero for all designs across the 1000 iterations of this simulation.

We also aim to evaluate the amount of person-time that participants are precluded from receiving a GLP1-RA because they are in the control arm of one of the potential RCTs. As shown in Supplementary Table 1, for **Designs 1 and 3**, we start by determining whether the results of RCT1 or of the hybrid RCT1-RWD analysis reject the null hypothesis. Because the simulated effect is zero, and we expect a truly negative effect of semaglutide versus standard-of-care on MACE based on the results of the SUSTAIN 6 trial [17], we shift the definition of the null hypothesis to be that the risk difference is a specified value larger than zero.

For the sake of this demonstration, we consider a significant result for superiority as an estimate of the risk difference with an upper 95% CI limit less than positive 1.1%. This value was chosen because a trial of 9500 participants (similar to our simulated RCT2) would be expected to have power of 0.8 to detect a risk difference of -1.1% (using $\alpha=0.05$). If we view the simulated data as having simulated CRD values that are shifted 1.1% more positive than the value of the CRD that our superiority trial would be powered to detect, then shifting standard criteria for superiority by the same amount would cause us to conclude that a result was significant if the estimated upper bound on the 95% confidence interval were less than positive 1.1%. Power to detect the true effect of zero is then calculated as the proportion of iterations in which the upper and lower confidence interval bounds were less than 1.1%. For the naïve pooled analysis of the simulated RCT1 and RWD in particular, it is possible that bias away from the null would lead to rejection of the null hypothesis that the risk difference equals 1.1% in the positive direction, suggesting inferiority of semaglutide compared to placebo. In these cases, we do not run simulated RCT2.

Note that this method of evaluating significance should actually be slightly conservative given that in the simulated data, the probability of the outcome is approximately the PIONEER6 placebo arm rate (4.2%) in both arms, whereas if a negative risk difference had been simulated, the treatment arm outcome probability would have been less than 4.2%. For example, with the same sample size *N*, if the treatment arm probability of the outcome were 3%, the variance of the difference in sample proportions (*V1*) would be smaller than the variance if the treatment arm probability of the outcome were also 4.2% (*V2*):

$$V1\approx\frac{0.03\left( 1-0.03 \right)}{N} + \frac{0.042\left( 1-0.042 \right)}{N} = \frac{0.069}{N}$$

$$<\frac{0.080}{N} = \frac{0.042(1-0.042)}{N} + \frac{0.042(1-0.042)}{N} \approx V2 .$$

Finally, the person-time participants are prevented from receiving any GLP1-RA for each design in a single iteration of the simulation is calculated as described in Supplementary Table 1 below. We report the average amount of person-time during which participants were prevented from receiving a GLP1-RA across all iterations for each design. While the event-driven SOUL trial will actually run for closer to four years, we only include person-time required to evaluate the outcome for this proposed study: MACE by one year after baseline.

**Supplementary Table 1: Calculation of Person-Time prevented from Receiving a GLP1-RA for each Design**

| **Design** | **Calculation of person-time prevented from receiving a GLP1-RA** |
| --- | --- |
| 1 | 1. If RCT1 result rejects null hypothesis: 1 year x 1591.5 placebo arm participants = 1591.5 person-years 2. If RCT1 result does not reject null hypothesis:    1. 1 year x 1591.5 placebo arm participants from RCT1 + 1 year x 4750 placebo arm participants from RCT2 = 6341.5 person-years |
| 2 | 1. 1 year x 4750 placebo arm participants from RCT2 = 4750 person-years |
| 3 | 1. If hybrid RCT1-RWD result rejects null hypothesis^§^: 1 year x 1591.5 placebo arm participants from RCT 1 = 1591.5 person-years 2. If hybrid RCT1-RWD result does not reject null hypothesis:    1. 1 year x 1591.5 placebo arm participants from RCT1 + 1 year x 4750 placebo arm participants from RCT2 = 6341.5 person-years |

**Caption:** GLP1-RA: glucagon-like peptide-1 receptor agonist. RCT: randomized controlled trial. RWD: real-world data.

^§^RWD participants were not prevented from receiving a GLP1-RA by being in an RCT control arm and so are not included in the amount of person-time during which patients are prevented from receiving a GLP1-RA.

A simple Super Learner [18] library of candidate algorithms was used for the ES-CVTMLE to improve computational efficiency of this simulation. The outcome regression was estimated using logistic regression. The Super Learner for the censoring mechanism ($P(C=0|A,W))$ and treatment mechanism ($P(A=1|W))$ for the combined RCT and RWD considered either a logistic regression or the sample mean. Because censoring was negligible in the RCT, the censoring mechanism in the RCT used within the ES-CVTMLE estimator only considered the sample proportion of non-missing outcomes. R Statistical Software version 4.2.2 was used for all simulations [19].

**Appendix 5: Data generation and results for simulation in which bias has no effect on NCO**

We also include a simulation in which the bias term, *B*, has no effect on the NCO. This simulation is included as a worst-case scenario for how hybrid **Design 3** could perform under a complete violation of the assumption that the factors causing bias in the relationship between the treatment and the true outcome also cause bias in the relationship between the treatment and the NCO. The process for generating the data for this simulation is the same as in Appendix 4, except that

$$NCO \sim Bernoulli\left( p ={logit}^{-1}\left( -3.33 + 0.2*W1 - 0.4*W2 + U_{nco} \right) \right).$$

Supplementary Figure 1 shows the results of 1000 iterations of this simulation both when the ES-CVTMLE uses the estimated average treatment effect on the NCO as an estimate of the causal gap and when the ES-CVTMLE only estimates bias based on the method described in Appendix 3, without considering the NCO.

**Supplementary Figure 1: Simulation Results by Study Design with Different Amounts of RWD Bias when Bias has No Effect on NCO**

**Caption:** RWD: real-world data. NCO: negative control outcome. GLP1-RA: glucagon-like peptide-1 receptor agonist. CI: Confidence Interval

1. **NCO bias estimate included b) NCO bias estimate not included**


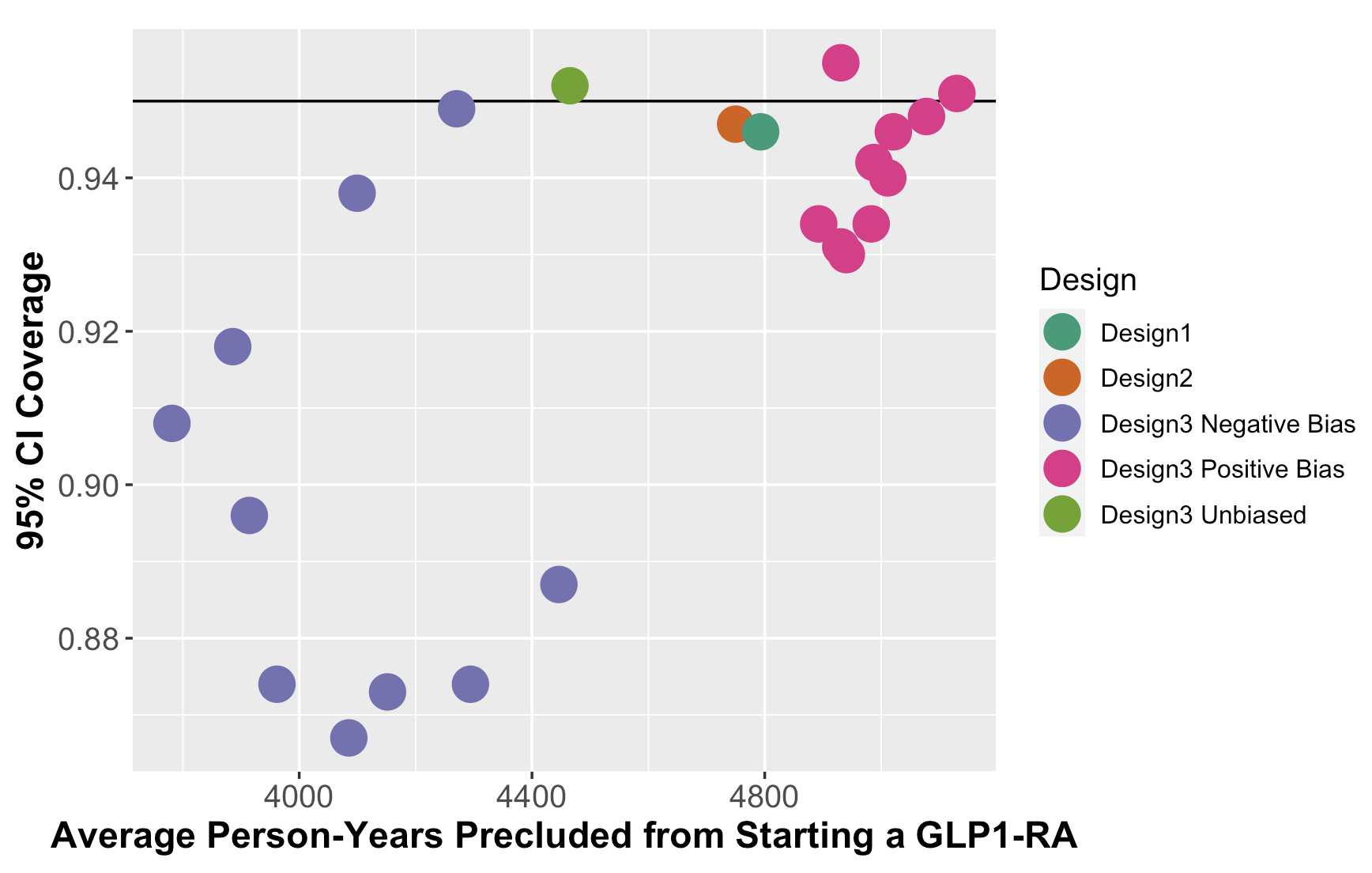

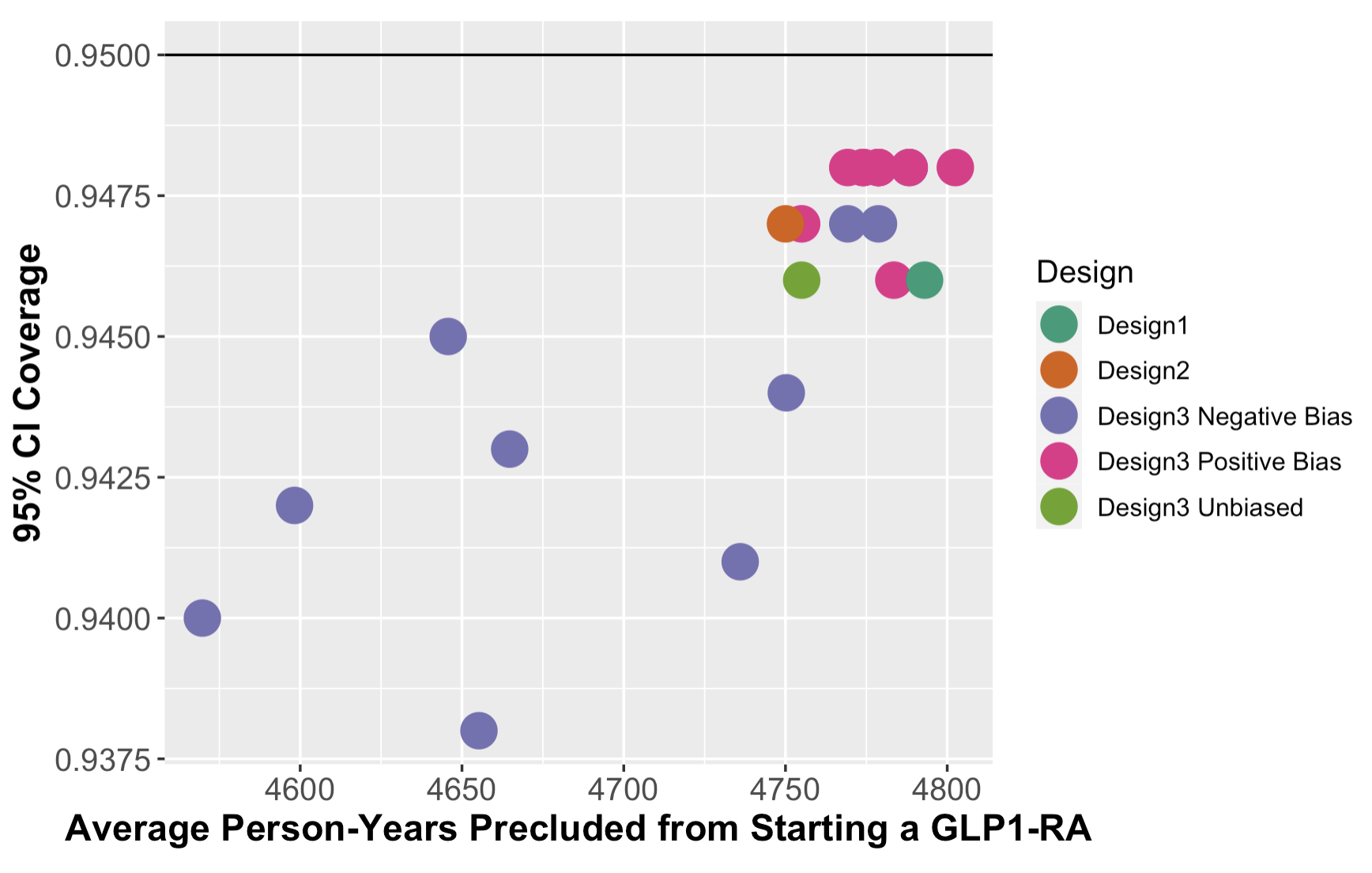


Because the bias term, *B*, has no effect on the NCO in this simulation, hybrid **Design 3** is more likely to incorporate biased RWD in either direction when the NCO is used to estimate bias. With bias in the positive direction (towards the null), the hybrid RCT-RWD design is less likely to reject the null hypothesis, leading to the follow-up RCT being run in more iterations. As a result, patient-time during which participants were precluded from receiving a GLP1-RA was larger on average for **Design 3** with positive bias than for **Designs 1** **and 2**.

With bias in the negative direction, **Design 3** led to as large as an average of 1012 fewer person-years during which participants could not start a GLP1-RA compared to **Design 1** but had 95% CI coverage ranging from 0.867 to 0.949. These results demonstrate the importance of choosing a good negative control outcome if this particular study design and estimator are selected.

If an appropriate negative control outcome is not available, the ES-CVTMLE may also only use one estimate of bias, as described in Appendix 3. In this context, the ES-CVTMLE is more conservative (less likely to include RWD), with coverage not less than 0.938 for any magnitude of bias, but a maximum average decrease in patient-years during which a GLP1-RA may not be prescribed of 223. The possibilities described in Supplementary Figure 1 should also be considered in the process of study design and estimator selection. Also note that the performance of **Design 1** compared to **Design 2** is slightly different in this simulation compared to the simulation in the main text. Such variability is expected when the same simulation is run different times although may be decreased by running more iterations.

**Appendix 6: Further details regarding specification of the ES-CVTMLE and unadjusted estimators**

The ES-CVTMLE estimator for the real data analysis used twenty cross-validation folds. The Super Learner libraries for the relevant regressions consisted of logistic regression or the sample mean for the outcome, logistic regression [20], a general additive model [21], or multivariate adaptive regression splines [22] for the propensity score, logistic regression [20], a general additive model [21], multivariate adaptive regression splines [22] or the sample mean for the RWD outcome censoring mechanism, and the sample mean only for the RCT outcome censoring mechanism. Because estimates vary somewhat when different random seeds are used to define cross-validation folds and train machine learning algorithms, the ES-CVTMLE estimator was run ten times with different random seeds, and the point estimate and upper and lower confidence interval bounds were averaged across these ten iterations.

Because censoring was negligible (0.3%) in the simulated and real data RCTs, a complete case analysis was conducted for the components of each study design that only involved RCT data.

**Appendix 7: Details for the construction of the CDM cohort**

Supplementary Tables 2-7 in this section describe how we translated the PIONEER 6 protocol into a format that could be implemented using the RWD. To minimize positivity issues, the cohort was trimmed to only include individuals with values of age, eGFR, HbA1c, HDL and LDL that fell within the 2.5-97.5% range of the values observed in PIONEER 6.

**Supplementary Table 2. Baseline Characteristics**

|  |  | **Comments** | |
| --- | --- | --- | --- |
|  | **Age** | Continuous | |
|  | **Sex** | Binary | |
|  | **Race** | To avoid positivity issues resulting from many small categories of baseline confounding variables, we categorized race as:  1. White  2. Black  3. Other  However, the “other” group is quite different between PIONEER 6 (more Asian participants) and CDM (more Hispanic participants). Asian participants in PIONEER 6 were more likely to live in Asia, whereas Asian participants in CDM were living in the United States. | |
| **Laboratory Measurements** | | We used the most recent measurement within six months prior to time zero. If there was more than one measurement on that day, we took an average of those measurements. We also filtered out measurements outside trustworthy ranges based on standard data cleaning rules. | |
|  | **HbA1c** | Continuous measurement | |
|  | **HDL** | Continuous measurement | |
|  | **LDL** | Continuous measurement | |
|  | **eGFR** | We used the MDRD calculation in CDM, but PIONEER 6 uses CKD-epi for calculating eGFR. | |
| **Medical history** | | Recorded any time prior to time zero, in any diagnosis position, and in either the in- or out-patient setting | |
|  | **Prior MI** | **ICD9**  Acute MI: 410.xx, Old MI: 412.xx | **ICD10**  MI: I21.X, I22.X  Old MI: I25.2 |
|  | **Prior stroke or TIA** | **ICD9**  431, 433.x1, 434.x1, 435.xx | **ICD10**  Stroke: I61.X, I63.X,  Old stroke: I69.1, I69.3, G45.X |
|  | **Prior heart failure** | **ICD9**  428.x, 402.01, 402.11, 402.91, 404.01, 404.11, 404.91, 404.03, 404.11, 404.13, 404.91, 404.93 | **ICD10**  I11.0, I13.0, I13.2, I50.X |
| **Drug history** | | Any claim with the specific medication in the six months prior to time zero | |
|  | **Cardiovascular medication** | Cardiovascular medication included the following subgroups: antihypertensives, lipid-lowering, anti-thrombosis, diuretics | |
|  | **T2DM medication** | A combination of any of the following: metformin, sulfonylureas (SU), thiazolidinediones (TZD), sodium-glucose transport protein 2 inhibitors (SGLT2-i). | |
|  | **Insulin** | Any insulin | |
| **Other** | |  | |
|  | **Morbid obesity** | This is based on ICD-9 and ICD-10 codes as there are only a limited number of BMI measurements in CDM.  ICD9: 278.01, ICD10: E66.01, E66.2 | |

**Caption:** CDM: Clinformatics® Data Mart Database. HbA1c: glycated hemoglobin. HDL: high-density lipoprotein. LDL: low-density lipoprotein. eGFR: estimated glomerular filtration rate. MDRD: modification of diet in renal disease. CKD-epi: chronic kidney disease epidemiology collaboration. MI: myocardial infarction. ICD: international statistical classification of diseases and related health problems. TIA: transient ischemic attack. T2DM: Type 2 diabetes mellitus. BMI: body mass index.

**Supplementary Table 3. AHFS codes for medications**

| **Grouping for drug history** | **Detailed grouping** | **AHFS** |
| --- | --- | --- |
| **Insulin** | All insulin | 68:20.08 |
|  | Long acting (including intermediate acting, human insulin and premix) | 68:20.08.08, 68:20.08.12, 68:20.08.16 |
|  | Short acting | 68:20.08.04 |
| **T2DM medication excluding insulin** | All T2DM medications excluding insulin | 68:20 but without:  68:20.08 (Insulins) |
|  | Metformin | 68:20.04 |
|  | TZD (rosiglitazone, pioglitazone) | 68:20.28 |
|  | SU | 68:20.20 |
| **CV medication** |  |  |
|  | Antithrombotic (antiplatelet and anticoagulants) | 20:12.04.08  20:12.04.12  20:12.04.14  20:12.18  20:12.20 |
|  | Antihypertensives | 24:08 (minus diuretics 24:08.24)  24:12  24:20  24:24  24:28  24:32 |
|  | Diuretics (pooled with antihypertensives) | 24:08.24 |
|  | Lipid lowering | 24:06.04,  24:06.05,  24:06.06,  24:06.08,  24:06.24 |

**Caption:** AHFS: American Hospital Formulary Service. T2DM: Type 2 diabetes mellitus. TZD: thiazolidinediones. SU: sulfonylureas. CV: cardiovascular.

**Supplementary Table 4. Primary MACE outcome in the PIONEER 6 trial and the translation to CDM**

| **PIONEER-6 trial definitions** | **ICD-9** | **ICD-10** |
| --- | --- | --- |
| First occurrence of  · Nonfatal myocardial infarction (MI)  · Nonfatal stroke  · Death from all cause | First occurrence measured in diagnosis position 1 and inpatient care setting | |
|  | **MI**: 410.X  **Stroke**: 431.X, 433.X1, 434.X1  **Mortality**: All cause death | **MI**: I21.X, I22.X  **Stroke**: I61.X, I63.X  **Mortality**: All cause death |

**Caption:** MACE: major adverse cardiovascular events. CDM: Clinformatics® Data Mart Database. ICD: international statistical classification of diseases and related health problems.

**Supplementary Table 5. Inclusion criteria** **in the PIONEER 6 trial and the translation to CDM**

| **PIONEER 6 inclusion criteria** | **ICD9** | **ICD 10** |
| --- | --- | --- |
| Informed consent | **N/A** |  |
| Men or women with type 2 diabetes | Our CDM cohort only includes individuals with diabetes.  Excluded patients with type 1 diabetes (T1D):  Measured at any time in any diagnosis position and inpatient or outpatient care setting:  T1D (ICD-9 250.x1, ICD-9 250.x3) | E10.X, O24.0 |
| Either of the following: |  |  |
| **Age ≥50 years at screening and at least one of the following conditions:** | Age ≥ 50 at time zero AND at least one of the following measured prior to time zero in any diagnosis position and inpatient or outpatient care setting: |  |
| Prior MI | Acute MI: 410.xx, Old MI: 412.xx | MI: I21.X, I22.X  Old MI: I25.2 |
| Prior stroke or TIA | Stroke or TIA: 431, 433.x1, 434.x1, 435.xx | Stroke: I61.X, I63.X,  Old stroke: I69.1, I69.3  TIA G45.X |
| Prior coronary, carotid or peripheral arterial revascularization including:  Percutaneous transluminal coronary angioplasty (PTCA)  Coronary artery bypass graft (CABG) | Coronary revascularization (PTCA, stenting, CABG)  PTCA:  ICD-9 proc: 00.66, 36.01, 36.02, 36.03, 36.05, 36.09  Stenting:  ICD-9 proc: 36.06, 36.07  CABG:  ICD-9 proc: 36.1x, 36.2  Transmyocardial revascularization:  ICD-9 proc: 36.31-36.34 | ICD-10-PCS: 027.X, 021.X |
| >50% stenosis on angiography or imaging of coronary, carotid, or lower  extremity arteries | Other forms of chronic ischemic heart disease  414.x  Peripheral vascular disease:  440.20 – 440.24, 440.29 – 440.32, 440.3, 440.4, 443.9 | Chronic ischemic heart disease: I25.X    Peripheral vascular disease:  I70.X, I73.9 |
| History of symptomatic coronary heart disease documented by positive exercise stress test or any cardiac imaging or unstable angina with ECG changes | N/A | |
| Asymptomatic cardiac ischemia documented by positive nuclear imaging test, exercise test or dobutamine stress echo | N/A | |
| Chronic heart failure NYHA class II-III | Heart Failure:  428.x, 402.01, 402.11, 402.91, 404.01, 404.11, 404.91, 404.03,404.11, 404.13, 404.91,404.93 | I11.0, I13.0, I13.2, I50.X |
| Moderate renal impairment (estimated glomerular filtration rate [eGFR] 30 to 59 ml/min/1.73 m^2^) | Chronic Kidney Disease (CKD) stage 3:  585.3 | CKD stage 3  N18.3 |
| **Age ≥60 years at screening and at least one of the following risk factors:** | Age ≥ 60 at time zero AND measured prior to time zero in any diagnosis position and inpatient or outpatient care setting: |  |
| Microalbuminuria or proteinuria | Proteinuria:  791.0  Albumin abnormality:  790.99 | Proteinuria:  R18.X  Albumin abnormality:  R77.0 |
| Hypertension and left ventricular hypertrophy by ECG or imaging | N/A | |
| Left ventricular systolic or diastolic dysfunction by imaging | N/A | |
| Ankle-brachial index <0.9 | Atherosclerosis of native arteries of the extremities with intermittent claudication:  440.21 | Atherosclerosis of native arteries of the extremities with intermittent claudication:  I70.21 |

**Caption:** CDM: Clinformatics® Data Mart Database. ICD: international statistical classification of diseases and related health problems. MI: myocardial infarction. TIA: transient ischemic attack. ECG: electrocardiogram. NYHA: New York Heart Association.

### **Supplementary Table 6. Exclusion criteria** **in the PIONEER 6 trial and the translation to CDM**

| **PIONEER 6 exclusion criteria** | **ICD-9** | **ICD-10** |
| --- | --- | --- |
| Known or suspected hypersensitivity to the trial product or related products. | N/A | |
| Previous participation in this trial. Participation is defined as signed informed consent | N/A | |
| Females of childbearing potential who are pregnant, breast-feeding or intend to become pregnant or are not using adequate contraceptive methods (adequate contraceptive measures as required by local law or practice) | N/A | |
| Receipt of any investigational medicinal product within 90 days before screening. | N/A | |
| Participation in another clinical trial of an investigational medicinal product. Participation in a clinical trial which evaluates stent(s) is allowed. | N/A | |
| Current or previous (within 90 days prior to screening) treatment with any GLP-1 receptor agonist, DPP-4 inhibitor or pramlintide | **Dispensing of at least one of the following medications in the 90 days prior to index date:**  Use of a GLP-1 receptor agonist ("exenatide", "liraglutide", "lixisenatide", "albiglutide", "dulaglutide", "semaglutide", "beinaglutide")  **or**  pramlintide  **or**  any dipeptidyl peptidase 4 (DPP-4) inhibitor ("sitagliptin", "vildagliptin", "saxagliptin", "alogliptin", "linagliptin", "gemigliptin", "evogliptin", "teneligliptin") | |
| Any disorder, which in the investigator’s opinion might jeopardize subject’s safety or compliance with the protocol. | N/A | |
| Family or personal history of multiple endocrine neoplasia type 2 (MEN 2) or medullary thyroid carcinoma (MTC) | Measured any time prior to time zero in any diagnosis position and inpatient or outpatient care setting:  MEN Type IIA:  258.02  MEN Type IIB:  258.03 | MEN Type IIA: E31.22  MEN, Type IIB: E31.23 |
| History of pancreatitis (acute or chronic). | Measured any time prior to time zero in any diagnosis position and inpatient or outpatient care setting:  Acute pancreatitis:  577.0  Chronic pancreatitis:  577.1 | K85, K86.0, K86.1 |
| History of major surgical procedures involving the stomach potentially affecting absorption of trial product (e.g., subtotal and total gastrectomy, sleeve gastrectomy, gastric bypass surgery). | Measured any time prior to time zero in any diagnosis position and inpatient or outpatient care setting:  Partial gastrectomy:  43.5x-43.8x  Total gastrectomy:  43.9x  Sleeve gastrectomy:  43.82  Gastric bypass:  44.3x,44.68, 44.95, 44.96, 44.97, 44.99, 44.5 | Bypass of stomach  0D16    Excision/resection of stomach  0DB6, 0DT6 |
| Subjects presently classified as being in New York Heart Association (NYHA) Class IV heart failure. | N/A | |
| Planned coronary, carotid or peripheral artery revascularization known on the day of screening | N/A | |
| Any of the following: myocardial infarction, stroke or hospitalization for unstable angina or transient ischemic attack within the past 60 days prior to screening. | Measured 60 days prior to time zero in any diagnosis position and inpatient or outpatient care setting:  MI, Stroke  Acute MI:  410.xx  Stroke:  431, 433.x1, 434.x1  Measured 60 days prior to time zero in any diagnosis position and inpatient care setting:  TIA:  435.xx  Unstable angina:  ICD9: 411.1 | **MI:** I21.X, I22.X  **Stroke:** I61.X, I63.X    Unstable angina:  ICD10: I20.0, I25.110, I25.700, I25.710, I25.720    TIA: G45 |
| Chronic or intermittent hemodialysis or peritoneal dialysis or severe renal impairment  (corresponding to eGFR <30 mL/min/1.73 m2). | Measured any time prior to time zero in any diagnosis position and inpatient or outpatient care setting:  CKD stage 4:  585.4  CKD stage 5:  585.5  ESRD:  585.6  Measured any time prior to time zero in any diagnosis position and inpatient or outpatient care setting at least twice:  Hemodialysis:  ICD9Proc: 39.95  Peritoneal dialysis:  ICD9Proc: 54.98 | CKD stage 4:  N18.4  CKD stage 5:  N18.5  ESRD:  N18.6    Hemodialysis:  5A1D    Peritoneal dialysis  3E1M    Trimming handles eGFR already. |
| History or presence of malignant neoplasms within the last 5 years (except basal and squamous cell skin cancer and carcinoma in situ). | Measured at any time prior to time zero in any diagnosis position and inpatient or outpatient care setting  History of malignant neoplasm:  140.xx-208.xx (except 173.xx, non-melanoma skin cancer) | C01-C99 except C44 |
| History of diabetic ketoacidosis. | Measured at any time prior to time zero in any diagnosis position and inpatient or outpatient care setting:  Secondary diabetes mellitus with ketoacidosis:  249.1  Diabetes with ketoacidosis:  250.1 | E08.1  E11.1  E13.1 |
| Proliferative retinopathy or maculopathy requiring acute treatment. Verified by fundus photography or dilated fundoscopy performed within 90 days prior to screening or within the period between screening and randomization. | N/A (due to difficulty in identifying acute treatment) |  |

**Caption:** CDM: Clinformatics® Data Mart Database. ICD: international statistical classification of diseases and related health problems. GLP-1: glucagon-like peptide-1. DPP-4: dipeptidyl peptidase-4. MI: myocardial infarction. TIA: transient ischemic attack. eGFR: estimated glomerular filtration rate. CKD: chronic kidney disease. ESRD: end-stage renal disease.

**Supplementary Table 7. Negative control outcome in the PIONEER 6 trial and the translation to CDM**

| **PIONEER-6 trial definitions** | **ICD-9** | **ICD-10** |
| --- | --- | --- |
| First occurrence of fracture | First occurrence measured in diagnosis position 1 and inpatient care setting | |
|  | 800-829 | S02**,** S12**,** S22**,** S32**,** S42**,** S52**,** S62**,** S72**,** S82**,** S92 |

**Caption:** CDM: Clinformatics® Data Mart Database. ICD: international statistical classification of diseases and related health problems.

**Appendix 8: References for Appendices**

1. Husain M, Birkenfeld AL, Donsmark M, et al. Oral semaglutide and cardiovascular outcomes in patients with type 2 diabetes. *N Engl J Med*. 2019;381(9):841-851. doi:10.1056/NEJMoa1901118

2. Neyman J. Sur les applications de la theorie des probabilites aux experiences agricoles: Essai des principes. English translation by D.M. Dabrowska and T.P. Speed (1990). *Stat Sci*. 1923;5:465-480.

3. Rubin DB. [On the application of probability theory to agricultural experiments. Essay on principles. Section 9.] Comment: Neyman (1923) and causal inference in experiments and observational studies. *Stat Sci*. 1990;5(4). doi:10.1214/ss/1177012032

4. Dang LE, Tarp JM, Abrahamsen TJ, et al. A Cross-Validated Targeted Maximum Likelihood Estimator for Data-Adaptive Experiment Selection Applied to the Augmentation of RCT Control Arms with External Data. arXiv. Preprint posted online October 11, 2022. arXiv:2210.05802

5. Robins J. A new approach to causal inference in mortality studies with a sustained exposure period—application to control of the healthy worker survivor effect. *Math Model*. 1986;7(9-12):1393-1512. doi:10.1016/0270-0255(86)90088-6

6. Pearl J. *Causality: Models, Reasoning, and Inference*. 2nd ed. Cambridge University Press; 2009. doi:10.1017/CBO9780511803161

7. Ghadessi M, Tang R, Zhou J, et al. A roadmap to using historical controls in clinical trials – by Drug Information Association Adaptive Design Scientific Working Group (DIA-ADSWG). *Orphanet J Rare Dis*. 2020;15(1):69. doi:10.1186/s13023-020-1332-x

8. Dahabreh IJ, Haneuse SJPA, Robins JM, et al. Study designs for extending causal inferences from a randomized trial to a target population. *Am J Epidemiol.* 2021; 190(8): 1632-1642. https://doi.org/10.1093/aje/kwaa270

9. U.S. Food and Drug Administration. Type 2 Diabetes Mellitus: Evaluating the Safety of New Drugs for Improving Glycemic Control: Guidance for Industry, (https://www.fda.gov/media/135936/download). 2020. Accessed April 25, 2023.

10. Franklin JM, Patorno E, Desai RJ, et al. Emulating randomized clinical trials with nonrandomized real-world evidence studies: First results from the RCT DUPLICATE initiative. *Circulation*. 2021;143(10):1002-1013. doi:10.1161/CIRCULATIONAHA.120.051718

11. Chiu YH, Dahabreh IJ. Selection on treatment in the target population of generalizabillity and transportability analyses. arXiv. Preprint posted online September 19, 2022. arXiv:2209.08758

12. Hernan MA. Estimating causal effects from epidemiological data. *J Epidemiol Community Health*. 2006;60(7):578-586. doi:10.1136/jech.2004.029496

13. Petersen ML, Porter KE, Gruber S, Wang Y, van der Laan MJ. Diagnosing and responding to violations in the positivity assumption. *Stat Methods Med Res*. 2012;21(1):31-54. doi:10.1177/0962280210386207

14. van der Laan MJ, Rose S. *Targeted Learning*. Springer New York; 2011. doi:10.1007/978-1-4419-9782-1

15. van der Laan MJ, Rubin D. Targeted maximum likelihood learning. *Int J Biostat*. 2006;2(1). doi:10.2202/1557-4679.1043

16. Lipsitch M, Tchetgen Tchetgen E, Cohen T. Negative controls: A tool for detecting confounding and bias in observational studies. *Epidemiology*. 2010;21(3):383-388. doi:10.1097/EDE.0b013e3181d61eeb

17. Marso SP, Bain SC, Consoli A, et al. Semaglutide and cardiovascular outcomes in patients with type 2 diabetes. *N Engl J Med*. 2016;375(19):1834-1844. doi:10.1056/NEJMoa1607141

18. van der Laan MJ, Polley EC, Hubbard AE. Super learner. *Stat Appl Genet Mol Biol*. 2007;6(1). doi:10.2202/1544-6115.1309

19. R Core Team. R: A language and environment for statistical computing. 2022. https://www.R-project.org/

20. Enea M. speedglm: Fitting Linear and Generalized Linear Models to Large Data Sets. 2021. https://CRAN.R-project.org/package=speedglm

21. Hastie T. gam: Generalized Additive Models. 2020. https://CRAN.R-project.org/package=gam

22. Milborrow S. earth: Multivariate Adaptive Regression Splines. 2020. https://CRAN.R-project.org/package=earth
